# Electronic Longitudinal Alcohol Study in Communities (ELAStiC) Wales – protocol for platform development

**DOI:** 10.23889/ijpds.v4i1.581

**Published:** 2019-05-20

**Authors:** L Trefan, A Akbari, S Paranjothy, DM Farewell, A Gartner, D Fone, J Greene, A Evans, A Smith, V Adekanmbi, J Kennedy, RA Lyons, SC Moore

**Affiliations:** 1 Division of Population Medicine, School of Medicine, Cardiff University, 3rd Floor Neuadd Meirionnydd, Heath Park, Cardiff CF14 4YS; 2 Health Data Research UK Wales and Northern Ireland, Swansea University Medical School, Singleton Park, Swansea SA2 8PP; 3 Crime and Security Research Institute and School of Dentistry, Cardiff University, Cardiff, CF14 4XY

## Abstract

**Introduction:**

Excessive alcohol consumption has adverse effects on health and there is a recognised need for the longitudinal analysis of population data to improve our understanding of the patterns of alcohol use, harms to consumers and those in their immediate environment. The UK has a number of linkable, longitudinal databases that if assembled properly could support valuable research on this topic.

**Aims and Objectives:**

This paper describes the development of a broad set of cross-linked cohorts, e-cohorts, surveys and linked electronic healthcare records (EHRs) to construct an alcohol-specific analytical platform in the United Kingdom using datasets on the population of Wales.

The objective of this paper is to provide a description of existing key datasets integrated with existing, routinely collected electronic health data on a secure platform, and relevant derived variables to enable population-based research on alcohol-related harm in Wales. We illustrate our use of these data with some exemplar research questions that are currently under investigation.

**Methods:**

Record-linkage of routine and observational datasets. Routine data includes hospital admissions, general practice, and cohorts specific to children. Two observational studies were included. Routine socioeconomic descriptors and mortality data were also linked.

**Conclusion:**

We described a record-linked, population-based research protocol for alcohol related harm on a secure platform. As the datasets used here are available in many countries, ELAStiC provides a template for setting up similar initiatives in other countries. We have also defined a number of alcohol specific variables using routinely-collected available data that can be used in other epidemiological studies into alcohol related outcomes. With over 10 years of longitudinal data, it will help to understand alcohol-related disease and health trajectories across the lifespan.

## Introduction

The excessive consumption of alcohol has adverse effects on health including liver cirrhosis [1], cancer [2], hypertension [3] and stroke [4]. There is also an increased risk of harm resulting from violence including homicide [5], suicide [6], road traffic accidents [7], domestic violence [8], and assault-related injury [9]. Alcohol use disorders and mental health disorders are often comorbid [10]. Key life events in adults (e.g. divorce, death of a partner) elicit stress [11] which in turn may promote alcohol use [12]. Estimates suggest alcohol misuse is accountable for 2.3 million premature deaths each year worldwide [13] plus many other non-fatal conditions [14, 15]. Half of those under 16 years of age report heavy episodic drinking [16] and excessive alcohol use is the third leading risk factor for disease and injury in Western Europe, the leading risk factor among 15 to 44 year olds globally [17].

In children, other than victimisation, most secondary harms associated with alcohol misuse do not figure in estimates of alcohol attributable fractions [15]. The number of children who are affected by parental alcohol misuse is largely unknown [18] although estimates suggest a third of all United Kingdom (UK) children live with at least one parent who uses alcohol hazardously [18]. How this impacts on their health, mental health and education is unclear. There may also be impacts on health service utilisation including contact with primary care, particularly out-of-hours services, completion of routine vaccinations and admissions to hospital.

Given the considerable costs to society of alcohol misuse and that it affects consumers directly as well as others in their environment, such as children, there is an identified need for research to improve our understanding of the causes and consequences of alcohol use. The UK is internationally preeminent in the quality and range of available longitudinal data collected to inform policy and practice [19, 20]. Longitudinal studies that follow individuals throughout their lives are well placed to improve our understanding of alcohol use patterns in communities, across the lifespan, and can illuminate plausible mechanisms promoting harm, knowledge of which can assist with the design and delivery of interventions [21, 22].

On this basis the UK’s Economic and Social Research Council, Medical Research Council and Alcohol Research UK funded research to exploit the availability of longitudinal data in the UK and address outstanding questions concerning the causes and consequences of alcohol use and misuse. The Electronic Longitudinal Alcohol Study in Communities (ELAStiC) project aims to leverage the value of a broad set of cohorts, e-cohorts, surveys and data linkage facilities to construct an alcohol-specific analytical platform to address key research aims within a UK Secure eResearch Platform (UKSeRP) [23]. The datasets selected for the research aims of the ELAStiC project have provided, or are designed to provide, key sources of evidence for social and health policy, and make substantial contributions to our understanding of disease and health trajectories across the life course induced by alcohol.

In this paper, focusing on data from the population of Wales, we provide a description of existing key datasets integrated with existing, routinely collected electronic health data on a secure platform, and relevant derived variables to enable population-based research on alcohol-related harm. We illustrate our use of these data with some exemplar research questions that are currently under investigation. This data resource has potential to support further research in this area, and inform plans for comparable analyses in different countries.

### Aims and Objectives

The research questions and the relevant ELAStiC project objectives in Wales are further detailed Table 1.

Aims and Objectives for the ELAStiC project

- incorporate data into the UKSeRP and develop facilities to enable research access.
- undertake hypothesis-driven research using this platform to provide critical insights into alcohol use, its effects and pathways into harm. The research questions on alcohol-related harm in Wales are shown in Table 1.
- make explicit the policy relevance of the work and exploit opportunities to interface with possible intervention development.

**Table 1: Research questions and detailed project objectives for the ELAStiC project in Wales.** 

**Q1: What is the effect on children’s health and educational achievement of living in households in which one or more adults have a defined alcohol-related harm to health?**

(1) To define each household included in the Welsh Electronic Cohort for Children (WECC) as an alcohol-problem household or not according to whether adults in each household have a recorded alcohol-related hospital admission, with or without a linked Accident & Emergency (A&E) attendance, or a general practitioner (GP) record of alcohol-related harm.
(2) To compare healthcare utilisation, hospital admissions for injuries and educational achievement between children living in an alcohol-problem household or not. This will require linkage of the child’s unique encrypted Anonymised Linkage Field (ALF) identifier to the linked anonymised Residential Anonymised Linkage Field (RALF) household identifier and subsequent to the adults ALFs within each RALF to extract the adult Patient Episode Database for Wales (PEDW) and Welsh Longitudinal General Practice (WLGP) data. We will then code each RALF (and respective child ALFs) as a ‘alcohol problem household’ yes/no, and the analysis will compare child outcomes between these two groups of households, adjusting for individual and household covariates and small area -Lower Super Output Area (LSOA)- covariates of multiple deprivation.

**Q2: What is the longitudinal relationship between alcohol consumption and physical and mental health outcomes in adults aged 18 to 74 years in Caerphilly county in Wales?**

(1) To extend the linkage of each anonymised adult subject in Caerphilly Health and Social Needs Study (E-CATALyST) to Welsh Demographic Service Data (WDSD), PEDW, Annual District Death Data (ADDE) and WLGP records to end-2015.
(2) To compare the 14-year risk of PEDW- and WLGP-recorded physical and mental health outcomes in adults associated with different levels of alcohol consumption at baseline.
(3) To compare the 7-year risk of physical and mental health survey outcomes in adults associated with change in reported consumption between baseline and wave two.
(4) To assess these risks specifically by age and sex, particularly in young adults aged 18-24 and 25-29 years, in males and females separately (if numbers permit).

**Q3: What are the trends in alcohol-related admissions in Wales over 16 years?**

To describe the 16-year trend in alcohol-related admissions in the Welsh adult population (16 years of age and over) by age, sex and socioeconomic position.

**Q4: What are the socioeconomic patterns in alcohol-related hospital admission in adults in Wales, considering individual alcohol consumption and other factors?**

(1) To define the study cohort and link data from Welsh Health Survey Data (WHSD) participants aged 16 and over, who consented to linkage, to WDSD, PEDW and ADDE data.
(2) To compare the risk of alcohol-related hospital admission between people living in more and less deprived circumstances, considering individual-level alcohol consumption including type of drink and smoking.

## Methods

### Data sources

#### SAIL Databank

The Secure Anonymised Information Linkage (SAIL) Databank at Swansea University contains health, social and education data on over three million residents of Wales, UK [24, 25]. It currently includes 14 core [26] and nine restricted core data sets [27] and contains over 10 billion records [28] (Table 2). Information governance for SAIL is overseen by an independent Information Governance Review Panel (IGRP) [24]. Core data stets can be accessed following IGRP approval; for access to restricted core datasets, permission from the data providers is required in additional to IGRP approval. Robust policies, structures, controls and special software are in place to protect privacy through a reliable matching, anonymisation and encryption process achieved in conjunction with the National Health Service Wales Informatics Service (NWIS) [29] using a split file approach [24, 25]. For each data set within the SAIL Databank, each included individual is assigned an Anonymised Linking Field (ALF) that enables cross-linking. The ALF is based on an individual’s National Health Service (NHS) number or a combination of unique identifiers such as name, gender and date of birth [28]. The smallest geographical area for which data are already linked and may be released from the SAIL Databank, after disclosure control to take account such as small numbers, is the Lower Super Output Area (LSOA) [30]. The LSOA codes can be used to link to reference data such as deprivation scores including the Townsend scores [31] and Welsh Index of Multiple Deprivation (WIMD 2008) [32] based on the LSOAs of the 2001 Census [33]. LSOAs can be classified into different Office for National Statistics (ONS) settlement types [34] such as: village - ‘village, hamlet and isolated dwellings –sparse/less sparse’; town - ‘town and fringe –sparse/less sparse’; and urban - ‘urban >10k -sparse/less sparse’. In the SAIL Databank different version of LSOAs (e.g. LSOAs of 2011 Census) and related deprivation scores and settlement types can be used but for the ELAStiC project the herein described ones were chosen because these were the most suitable for the later describe datasets.

**Table 2: Secure Anonymised Information Linkage (SAIL) Databank core- and restricted core datasets.** Source: https://saildatabank.com/saildata/sail-datasets/#core Source: https://saildatabank.com/saildata/sail-datasets/#core-restricted Note: the ELAStiC data system includes a subset of these data as described in the text

CORE Annual District Birth Extract Annual District Death Extract (ADDE) Diagnostics & Therapy Services Waiting Times Emergency Department Data Clinical Care Dataset National Community Health Database Outpatient Outpatient Referral Patient Episode Database for Wales (PEDW) Referral to Treatment Times Postponed Admitted Procedures Primary Care GP (Audit+) (WLGP) UK Health Dimensions Welsh Demographic Service (WDSD)

CORE-RESTRICTED Active Adult Survey Bowel Screening Wales Breast Test Wales Cervical Screening Wales Congenital Anomaly Register and Information Services for Wales Education Attainment National Survey for Wales Welsh Cancer Intelligence Surveillance Unit Welsh Health Survey (WHSD)

The SAIL Databank includes anonymised identifiers for all households in Wales and those allow household-level data from local authorities and others to be linked with individual health-related data. This linkage uses ALFs and an additional Residential Anonymised Linking Field (RALF). Address data are matched at NWIS where identifiable addresses are replaced with RALFs [35]. The residence-based metrics are then fully incorporated into the SAIL Databank by linking RALFs to ALFs, so this way a person can be related to household environmental exposure.

#### ADDE

The Annual District Death Extract (ADDE) [36] is maintained by the ONS and contains death registration data for Welsh residents (those who died outside of Wales as well) including the underlying cause of death using the International Statistical Classification of Disease and Related Health Problems codes (ICD-10) [37]. These data are provided in an anonymised form to SAIL by NWIS.

#### WDSD

The Welsh Demographic Service Dataset (WDSD) is maintained by NWIS, and contains addresses and registration history for all individuals who register with a general practitioner (GP) in Wales. Dates for each address record update are retained, thereby providing durations of residency across different homes. This creates the opportunity for detailed exposure history, by linking to local environment exposures at each address for each individual [38]. These data are used to track population migration and record length of exposure for individuals within linked datasets.

#### PEDW

The Patient Episode Database for Wales (PEDW) includes demographic and clinical data on all inpatient and day case admissions in NHS Wales hospitals and on all Welsh residents treated in England. Very sensitive data such as human immunodeficiency virus (HIV) are removed from PEDW before release. Each record of an admission contains fields that include, among others: date of admission; admission method (e.g. emergency or elective); episode and spell number; provider unit code; specialty code; patient classification (inpatient or day case); 14 diagnoses codes based on ICD-10 codes [37]; and six procedure code fields using the Office of Population, Censuses and Surveys Classification of Surgical Operations and Procedures version 4.8 (OPCS-4.8) [39] by date; discharge destination (to identify inter-hospital transfers); discharge method (to identify death in hospital); and date of discharge [40]. The pseudo-anonymisation process results in the encryption of a unique ALF that enables a patient-based analysis rather than an admissions-based analysis. Each PEDW record is also linked to the LSOA of residence and through the LSOA code is then can be linked to deprivation scores, which in this study are deciles and quintiles of WIMD 2008 [32] and deciles of Townsend scores [31]. LSOAs were classified into three different (urban/town/rural) settlement types as well [34]. Admission can be therefore attributed to LSOA deprivation levels and settlement types in the analysis.

We defined a set of alcohol-relevant exposure and outcome variables using both diagnosis and procedure codes. The first group of variables were modified Charlson comorbidity scores [41], which predict 10-year survival in patients with multiple comorbidities. These variables include a general Charlson score, and its constituent elements with the exception of HIV as binary (yes/no) flags were defined. The general Charlson score was calculated as sum of the weights for each of the composite conditions (see Supplementary Appendix 1) in all secondary diagnosis fields (2-14 coding positions).

Alcohol-related hospital admissions were defined based on our previous work [42]. Briefly, these were two sets of variables based on ICD-10 codes for alcohol-related diagnoses. The first set of variables was defined based on alcohol-related diagnoses codes in any coding position, the second set of variables was defined based on the same diagnoses codes in the fourth position if the first three coding positions contain only R or Z codes (except for R78.0, Z50.2, Z71.4, Z72.1). We defined admissions for special infections and injuries using codes starting by ‘A’, ‘S’,’T’,’V’ (indicating gastro-intestinal infection, head injuries, poisoning, accidents) and their combinations present in any- or first occurrence coding position. The data were also flagged to denote hospital admission relating to each ICD-10 chapter. To do this two sets variables were defined, the first set for any position, the second set for first occurrence of the codes of the relevant ICD-10 chapter. Operation procedures flags were defined based on any code position in PEDW operation codes. In addition we have flagged admissions in children relating to victimisation and alcohol-related harms, e.g. assault, reduction of fracture or mandible. (Details are available Supplementary Appendix 1.)

#### WLGP

The Welsh Longitudinal General Practice (WLGP) dataset is the source of primary care data in the SAIL Databank and contains information about GP contacts (Read codes) for each registered individual in a SAIL Databank supplying general practice data. Read codes are the standard clinical terminology system used in general practices in the UK and it is regularly updated [43]. GPs enter medical diagnoses, symptoms and prescribed drugs using Read codes. The SAIL Databank currently receives data on consultations and prescriptions from approximately 75% of general practices in Wales [28].

We used Read codes to define a set of variables for denoting individual level alcohol consumption, alcohol-related illnesses and alcohol-problem households. Read codes version2 (V2) were downloaded from of Health and Social Care Information Centre website [44]. For V2 codes, 89,219 records were available with their description fields - these records covered all V2 symptom terminologies. These codes were imported into a spreadsheet (MSExcel 2010) and the keywords of ‘Alcohol’, ‘Alcoho’, ‘Alcoh’, ‘drink’ and ‘Teetotaller’ were searched in the three description fields. The resulting records were scrutinised by an experienced general practitioner and epidemiologist for relevance. Further searches were carried out for medications for alcoholism (‘Disulfiram’ and ‘Antabuse’) in the prescribed drug list. The final set of alcohol-related codes is shown in Supplementary Appendix 2.

We mapped the Read codes in GP data to ICD-10 chapters to create variables that denote health status for individuals, based on mapping tools available from the previously mentioned Health and Social Care Information Centre website [44]. The relation between the former and latter codes is one to many (1:N). This map was imported into a database management software (MS Access2010) and queries were run based on using the first character of the ICD-10 chapter (‘Like A*’). This procedure resulted in GP read code records grouped by ICD-10 chapter. These grouped records were exported to spreadsheets (MS Excel 2010) chapter by chapter. These exported records on spreadsheet was also scrutinised by the previously mentioned same clinical person and modification were made as necessary.

Read code based flags were also identified for common mental disorders (e.g. anxiety, depression) using a previously defined and verified algorithm [43], which uses both symptom- and (drug) prescription codes. Existing self-assessment based mental health variables (e.g. SF-36) [45] were also available.

#### WECC

The Wales Electronic Cohort for Children (WECC), a derived dataset that is a part of SAIL, comprises all children born in or living in Wales and registered with a GP in Wales between 1 Jan 1990 and 31 Dec 2013. WECC data contains pregnancy- and birth outcome variables such as maternal smoking, birth weight, multiple birth, stillbirth, congenital anomaly, breast feeding and child health interventions (immunisation) [46].

WECC is already record-linked to the National Pupil database and Pupil Level Annual School Census for education outcomes [47]. The educational data set contains assessment results for years 2003-2012, with sparse information for earlier years. Two assessments of educational attainment were used to answer the specified research questions in ELAStiC: Key stage 1 (KS1) is a national assessment in mathematics and in the English or Welsh language at age of six or seven years, and key stage 2 (KS2) is the equivalent national assessment at age of 10 or 11 years.

#### E-CATALyST

The Caerphilly Health and Social Needs Electronic Cohort (E-CATALyST) is a prospective cohort study of residents of Caerphilly county borough, Wales, UK [48]. Two surveys were carried out as part of E-CATALyST. In 2001 a stratified random sample of 22,236 individuals aged 18 and above resulted in 10,892 respondents providing valid information. In 2008 the survey was repeated with participants who were still residents of the borough. Of these, 4,558 provided data at both waves. The study has detailed information on a wide range of social, demographic and economic risk factors (e.g. age, gender, socioeconomic status, educational achievement, employment, household income, council tax band) as well as health and lifestyle outcome data (e.g. cardiovascular risk factors, limiting long-term illness). The survey results were stored in one SPSS file [49], which was uploaded to SAIL where the ALFs for linkage were created for participants.

#### WHSD

The Welsh Health Survey Dataset (WHSD) is an annual survey collected and maintained by Welsh Government. It provides information about the health of people living in Wales, the way they use health services, and their health-related lifestyle. It is based on a representative sample of people living in private households in Wales, selected using a random sample. It includes around 15,000 adults per year [50].

The survey includes questions on alcohol use on the heaviest drinking day in the past week, including the number of units and the type of drink consumed, as well as smoking and socio-demographic information.

## Data linkage of data sources

To answer the scientific question (Q1) children’s WECC core (v1.3), PEDW- and WLGP data were extracted between the first available date and 7-Oct-2012. In a separate procedure WDSD, PEDW, and WLGP data were used to identify hospital and general practice data indicating adults’ alcohol-related admissions and appointments. ALFs for those records were linked to the population data, which contains their anonymised household identification, and flagged as an ‘alcohol problem household’. Where household contained children, these data together with the children’s PEDW, WLGP data were linked to WECC data (Figure 1).

**Figure 1: Datasets, their relevant extraction dates and their linkage used in the Electronic Longitudinal Alcohol Study in Communities (ELAStiC) project.**
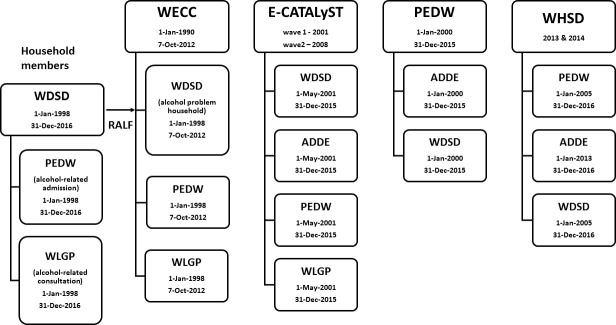
Note: WDSD: Welsh Demographic Dataset; PEDW: Patient Episode Database for Wales; WLGP: Welsh Longitudinal General Practice (data); RALF: Residential Anonymised Linking Field; WECC: Wales Electronic Cohort for Children; E-CATALyST: Caerphilly Health and Social Needs Electronic Cohort; ADDE: Annual District Death Extract; WHSD: Welsh Health Survey Dataset

For the E-CATALyST part of the project (answering Q2), PEDW and WLGP data were extracted between 1-May-2001 and 31-Dec-2105. E-CATALyST original data were linked to these and relevant WDSD and ADDE data (Figure 1).

To answer Q3, PEDW data were extracted between 1-Jan-2000 and 31-Dec-2015. These data together with relevant WDSD and ADDE data were linked (Figure 1).

To answer Q4, record-linked data for the survey years 2013 and 2014 were pooled. Around half of the respondents gave permission to link their answers to other routine data but the question on consent was only introduced during the survey year 2013. For the individuals who agreed (n=11,694) the survey data were linked to WDSD, ADDE and PEDW data, extracted between 1-Jan-2005 and 31-Dec-2016 (Figure 1). This resulted in successful linkage of records for 11,038 individuals, or 96.8% of the total (loss n=372). The period of data covered allowed consideration of historical information before the survey date.

## Discussion

A range of routinely-available datasets available in Wales have been integrated into the ELAStiC project on the SAIL Databank. These data can be used to address research questions concerning alcohol-related harm longitudinally. These datasets are population-based, and enhanced through record-linkage with data from a cohort study that was conducted in one Welsh county. The ELAStiC project residing in the SAIL Databank is therefore a population-based secure analytic platform with over ten years of longitudinal data and is able to further our understanding of alcohol-related disease and health trajectories across the life course.

When total-population data were used in this work, one important advantage of total-population approaches is that in a clear sense they maximise the power available to address a particular research question: if a total-population approach is unable to detect an interesting relationship between exposure and outcome, then the effect is arguably either undetectable or negligible.

Of the datasets to be used in the SAIL Databank, one was core restricted (WHSD), four were core (ADDE, PEDW, WLGP, WDSD), one was an e-cohort (WECC) and one was a project restricted survey (E-CATALyST).

Although individual datasets (e.g. PEDW) contain socioeconomic and mortality related variables, linkage to WDSD and ADDE datasets was required to provide an additional level of important detail on those factors not collected in routine EHRs, such as exact mortality details and change of residence. The addition of these details means that individuals who move, die, or households that change composition can be identified therefore define exposure over time correctly.

Of the GP Read codes used, symptom codes are mostly considered. Version 2 of these codes was used for defining different health status. There are other types of primary care classifications available such as Read code version 3 and SNOMED [44]. While the vast majority (97%) of the data used was in version 2, developments highlight the changing landscape of routine data and the need to ensure data is comparable over time.

PEDW data does not contain any data on sexually transmitted disease. The general Charlson comorbidity score described in this paper is therefore missing its HIV component [41], forming a modified version of the Charlson score which may be limited in further UK or other countries analyses where these data are available. Differences are likely to be relatively small: only around 1.1% of the Welsh population are diagnosed with HIV per year in the time periods used in this work.

Although the efficiency of SAIL Databank anonymisation and encryption process itself is above 99.8% [25], any record that has no ALF is ultimately a missing one because it cannot be linked. We assessed the extent of missing data in the linked datasets; for example, in the case of WHSD around half of respondents to the WHSD agreed to data linkage, resulting in the potential for those who agreed being systematically different to those who did not in terms of their alcohol consumption or other factors. By contrast, failure of linkage was very minimal (3.2%). The distribution by age and sex was similar to the total sample; one limitation is that the linked sample may not be representative of the population, an issue that must be considered in any analysis. Within the WHSD sample key demographic data were complete but there were missing responses to some of the individual survey questions, ranging from 0.6% for drinking frequency to 4.9% for BMI. Imputation is a natural option for analysis [51] and these percentages are low and unlikely to introduce bias in the analysis.

In the SAIL Databank deprivation and settlement type information can be linked only at LSOA level therefore these pieces of information cannot be used at person level in the analyses. Standard, robust policies for disclosure control also prevent public release of outputs in which any frequency of a variable was less than five. These reasonable limitations do nevertheless restrict what can be analysed and published using this system.

One further implication of disclosure control is the difficulty in satisfying the desire for fully replicable research. For obvious reasons, data cannot be made public in an unrestricted way; more subtle problems include the fact that, even within SAIL, it can be hard to confirm if exactly the same answers have been obtained by two independent groups of researchers.

Hospital admission data reflect the more serious alcohol-related cases, and therefore capture the more severe end of the alcohol-related harm spectrum. There may also be externalities that impact on the likelihood of admission, such as resources available to specialist alcohol teams. In this respect, the availability of GP data provides additional insights into the alcohol-harm trajectory as GPs are likely to provide the first clinical contact in many non-acute cases. GP data may also reveal ongoing care, medication and referrals following an admission.

One limitation of the approach described in this work (shared with most secondary analyses) is the difficulty in probing the validity of outliers or other unexpected patterns in the data. Lacking direct contact with the data-gatherer, the veracity of extreme observations can be difficult to determine, and requires error-prone judgments on the part of the analyst. Obvious examples include reports of particularly high alcohol consumption: this could be due to incorrect units, extra digits, survey hijinks, or a true reflection of that individual’s consumption.

There are general limitations to the use of routine linked data gathered primarily for the appropriate management of patients through healthcare systems. The realities of processing data that were not collected principally for the purposes of research means that hands-on experience of using these data systems, combined with advice from practicing healthcare professionals, is invaluable when undertaking analysis.

## Conclusion

An unprecedented level of representative data is available in Wales that offers opportunities to reveal the causes, consequences and mechanisms of alcohol-related harm across the lifespan. As the datasets used here are available in many countries, this protocol provides a template for setting up similar initiatives in other elsewhere.

### Research governance and ethics

Approval for the use of anonymised data in this study, provisioned within the Secure Anonymised Information Linkage (SAIL) Databank was granted by an independent Information Governance Review Panel (IGRP), with membership comprised of senior representatives from the British Medical Association (BMA), the National Research Ethics Service (NRES), Public Health Wales and NHS Wales Informatics Service (NWIS). Usage of WHSD data was approved by Welsh Government. The use of anonymised data for research is outside the scope of the EU General Data Protection Regulations (GDPR) and the UK Data Protection Act.

## Supplementary Appendices

Appendix 1: Describes variable definitions for alcohol-related hospital admissions of PEDW data.

Appendix 2: Describes GP Read codes defining alcohol-related GP consultations.

## Abbreviations

A&E	Accident and Emergency
ADDE	Annual District Death Extract
ALF	Anonymised Linking Field
E-CATALyST	Caerphilly Health and Social Needs Study
EHR	Electronic Health Records
ELAStiC	Alcohol Misuse: Electronic Longitudinal Alcohol Study in Communities
GP	General Practitioner
HIRU	Health Information Research Unit
HIV	Human Immunodeficiency Virus
ICD-10	International Statistical Classification of Disease and Related Health Problems
IGRP	Information Governance Review Panel
LSOA	Lower Super Output Area
NHS	National Health Service
NHS Wales	NHS in Wales
NWIS	NHS Wales Informatics Service
ONS	Office for National Statistics
PEDW	Patient Episode Data for Wales
RALF	Residential Anonymised Linking Fields
SAIL	Secure Anonymised Information Linkage (Databank)
UK	United Kingdom
UKSeRP	UK Secure eResearch Platform
WDSD	Welsh Demographic Service Dataset
WECC	Wales Electronic Cohort for Children
WHSD	Welsh Health Survey Dataset
WIMD	Welsh Index of Multiple Deprivation
WLGP	Welsh Longitudinal General Practice
