# Supplementary material for: Electronic Longitudinal Alcohol Study in Communities (ELAStiC) Wales – protocol for platform development
Source: Int J Popul Data Sci. 2019 May 20;4(1):581. doi: 10.23889/ijpds.v4i1.581 (PMC8142962; doi:10.23889/ijpds.v4i1.581)
Supplement: Appendix 1: Describes variable definitions for alcohol-related hospital admissions of PEDW data. [file ijpds-04-581-s001.pdf]

S1. Variables defined based on Patient Episode Database for Wales (PEDW) data using International Statistical Classification of Disease and Related Health Problems (ICD-10) codes.

Variable required	Description	ICD-10 codes	Position
CHARLSON_SCORE	Charlson comorbidity single score	Using CHARLSON_SC_FLAG_01 to CHARLSON_SC_FLAG_16 in 2-14 coding positions with individual weights	ANY
CHARLSON_SC_FLAG_01	Charlson score individual flag for acute myocardial infarction (Yes/No)	I21.0-I21.9, I22.0-I22.9, I25.2, I23.0-I23.8, I25.8	ANY
CHARLSON_SC_FLAG_02	Charlson score individual flag for cerebral-vascular accident (Yes/No)	G45.0, G45.1, G45.2, G45.4, G45.8, G45.9, G46.0-G46.8, I60.0-I60.9, I61.0-I61.9, I62.0-I62.9, I63.-I63.9, I64, I65.0-I65.9, I66.0-I66.9, I67.0, I67.1, I67.2, I67.4, I67.5, I67.6, I67.7, I6.78, I67.9, I68.1, I68.2, I68.8, I69.-I69.8, I67.3, I68.0	ANY
CHARLSON_SC_FLAG_03	Charlson score individual flag for congestive heart failure (Yes/No)	I50.0-I50.9	ANY
CHARLSON_SC_FLAG_04	Charlson score individual flag for connective tissue disorder (Yes/No)	M05.0-M05.9, M06.0, M06.3, M06.9, M32.0-M32.9, M33.2, M34.0-M34.9, M35.3	ANY
CHARLSON_SC_FLAG_05	Charlson score individual flag for dementia (Yes/No)	F00.0-F00.9, F01.0-F01.9, F02.0-F02.8, F05.1,F03	ANY
CHARLSON_SC_FLAG_06	Charlson score individual flag for diabetes (without long term complications) (Yes/No)	E10.1, E10.5, E10.9, E11.1, E11.5, E11.9, E13.1, E13.5, E13.9, E14.1, E14.5, E14.9, E10.6, E10.8, E11.6, E11.8, E13.6, E13.8, E14.6, E14.8	ANY

Variable required	Description	ICD-10 codes	Position
CHARLSON_SC_FLAG_07	Charlson score individual flag for diabetes (with long term complications) (Yes/No)	E10.2, E10.3, E10.4, E11.2, E11.3, E11.4, E13.2, E13.3, E13.4, E14.2, E14.3, E14.4, E10.7, E11.7, E13.7, E14.7	ANY
CHARLSON_SC_FLAG_08	Charlson score individual flag for mild or moderate liver disease (Yes/No)	K70.2, K70.3, K71.7, K73.0-K73.9, K74.0, K74.1,K74.2, K74.3, K74.4, K74.5, K74.6	ANY
CHARLSON_SC_FLAG_09	Charlson score individual flag for severe liver disease (Yes/No)	K72.1, K72.9, K76.6, K76.7	ANY
CHARLSON_SC_FLAG_10	Charlson score individual flag for peptic ulcer (Yes/No)	K25, K26, K27, K28	ANY
CHARLSON_SC_FLAG_11	Charlson score individual flag for peripheral vascular disease (Yes/No)	I71.0-I71.9, I73.9, I79.0, R02, Z95.8, Z95.9	ANY
CHARLSON_SC_FLAG_12	Charlson score individual flag for pulmonary disease (Yes/No)	J40, J41.0-J41.8, J42, J43.0-J43.9, J44.0-J44.9, J45.0-J45.9, J46, J47, J60, J61, J62.0-J62.8, J63.0-J63.8, J64, J65, J66.0-J66.8, J67.0-J67.9	ANY
CHARLSON_SC_FLAG_13	Charlson score individual flag for cancer (Yes/No)	C00.0-C76.8, C81.0-C85.9, C88.3, C88.7, C88.9, C90.0, C90.1, C91.0-C91.9, C92.0-C92.9, C93.0-C93.9, C94.0, C94.1, C94.2, C94.3, C94.5, C94.7, C95.0-C95.9, C96.0-C96.9, C80.0-C80.9, C88.0, C88.1, C88.2, C90.2, C94.4, C97	ANY
CHARLSON_SC_FLAG_14	Charlson score individual flag for metastatic cancer (Yes/No)	C77.0-C77.9, C78.0-C78.8, C79.0-C79.9	ANY

Variable required	Description	ICD-10 codes	Position
CHARLSON_SC_FLAG_15	Charlson score individual flag for paraplegia (Yes/No)	G04.1, G81.0-G81.9, G82.0, G82.1, G82.2	ANY
CHARLSON_SC_FLAG_16	Charlson Score individual flag for renal disease (Yes/No)	N01, N03, N05.2, N05.3, N05.4, N05.5, N05.6, N07.2, N07.3, N07.4, N18.1-N18.9, N19, N25.0-N25.9, I12.0-I12.9, I13.0-I13.9	ANY
PEDW_ALC_FLAG_01	Alcohol-related admission (Yes/No)	E24.4, E51.2, F10.0–F10.9, G31.2, G40.5, G62.1, G72.1, I42.6, K29.2, K70.0-K70.4, K70.9, K85.2, K86.0, O35.4, R78.0, T51.0, X45.0–X45.2, X45.4–X45.6, X45.8, X45.9, X65.0–X65.2, X65.4–X65.6, X65.8, X65.9, Y15.0, Y15.2, Y15.4, Y15.8, Y15.9, Y90.0-Y90.9, Y91.0–Y91.3, Y91.9, Z50.2, Z71.4, Z72.1	ANY/123RZ
PEDW_ALC_FLAG_03	F100 Acute intoxication(Yes/No)	F10.0	ANY/123RZ
PEDW_ALC_FLAG_04	F101 Harmful use(Yes/No)	F10.1	ANY/123RZ
PEDW_ALC_FLAG_05	F102 Dependence syndrome(Yes/No)	F10.2	ANY/123RZ
PEDW_ALC_FLAG_06	F103 Withdrawal(Yes/No)	F10.3	ANY/123RZ
PEDW_ALC_FLAG_07	F104 Withdrawal state(Yes/No)	F10.4	ANY/123RZ
PEDW_ALC_FLAG_08	Other F (F105, F106, F107, F108, F109) codes(Yes/No)	F10.5, F10.6, F10.7, F10.8, F10.9	ANY/123RZ

Variable required	Description	ICD-10 codes	Position
PEDW_ALC_FLAG_09	F10 Mental and behavioural disorders due to use of alcohol(Yes/No)	F10.0, F10.1, F10.2, F10.3, F10.4, F10.5, F10.6, F10.7, F10.8, F10.9	ANY/123RZ
PEDW_ALC_FLAG_10	K700 Alcoholic fatty liver(Yes/No)	K70.0	ANY/123RZ
PEDW_ALC_FLAG_11	K701 Alcoholic hepatitis(Yes/No)	K70.1	ANY/123RZ
PEDW_ALC_FLAG_12	K702 Alcoholic fibrosis and sclerosis of liver(Yes/No)	K70.2	ANY/123RZ
PEDW_ALC_FLAG_13	K703 Alcoholic cirrhosis of liver(Yes/No)	K70.3	ANY/123RZ
PEDW_ALC_FLAG_14	K704 Alcoholic hepatic failure(Yes/No)	K70.4	ANY/123RZ
PEDW_ALC_FLAG_15	K709 Alcoholic liver disease, unspecified(Yes/No)	K70.9	ANY/123RZ
PEDW_ALC_FLAG_16	K70 Alcoholic liver disease(Yes/No)	K70.0, K70.1, K70.2, K70.3, K70.4, K70.9	ANY/123RZ
PEDW_ALC_FLAG_17	K852 Alcohol-induced acute pancreatitis(Yes/No)	K85.2	ANY/123RZ
PEDW_ALC_FLAG_18	K860 Alcohol-induced chronic pancreatitis-<br>ANY (Yes/No)	K86.0	ANY/123RZ
PEDW_ALC_FLAG_19	T510 Toxic effect: Ethanol(Yes/No)	T51.0	ANY/123RZ

Variable required	Description	ICD-10 codes	Position
PEDW_ALC_FLAG_20	X45, X65 External cause of morbidity(Yes/No)	X45.0–X45.9, X65.0–X65.9	ANY/123RZ
PEDW_ALC_FLAG_21	Y15, Y90, Y91 External cause of morbidity(Yes/No)	Y15.0, Y15.2, Y15.4, Y15.8, Y15.9, Y90.0-Y90.9, Y91.0–Y91.3, Y91.9	ANY/123RZ
PEDW_ALC_FLAG_22	Z Alcohol use(Yes/No)	Z50.2, Z71.4, Z72.1	ANY/123RZ
PEDW_ALC_FLAG_23	Foetal alcohol syndrome(Yes/No)	Q86.0	ANY/123RZ
PEDW_ADD_FLAG_01	Acute upper respiratory tract infections (Yes/No)	J00–J06, J10.1, J10.8, J11.1, J11.8	ANY/1STNONRZ
PEDW_ADD_FLAG_02	Influenza And Pneumonia (Yes/No)	J09 – J18	ANY/1STNONRZ
PEDW_ADD_FLAG_03	Acute lower respiratory infections including bronchiolitis (Yes/No)	J20 – J22	ANY/1STNONRZ
PEDW_ADD_FLAG_04	Acute bronchiolitis (Yes/No)	J21	ANY/1STNONRZ
PEDW_ADD_FLAG_05	Asthma (Yes/No)	J45-J46	ANY/1STNONRZ
PEDW_ADD_FLAG_06	Injury (Yes/No)	S00-T19	ANY/1STNONRZ
PEDW_ADD_FLAG_07	Burns (Yes/No)	T20–T32, T33-T35	ANY/1STNONRZ
PEDW_ADD_FLAG_08	Toxic effects (Yes/No)	T36–T71, T74, T75, T79	ANY/1STNONRZ
PEDW_ADD_FLAG_09	Head injury (Yes/No)	S06.0, S02.0, S02.1, S02.2, S02.3, S02.7, S02.8, S06.1 to S06.9	ANY/1STNONRZ
PEDW_ADD_FLAG_10	External causes short list (Yes/No)	V01-V99	ANY/1STNONRZ

Variable required	Description	ICD-10 codes	Position
PEDW_ADD_FLAG_11	External causes long list (Yes/No)	V01-V99, W00-X59, X60-X84, X55-Y09, Y10-Y36	ANY/1STNONRZ
PEDW_ADD_FLAG_12	Gastro-intestinal infection (Yes/No)	A00-A09	ANY/1STNONRZ
PEDW_ADD_FLAG_13	Digestive (Yes/No)	K00-K93	ANY/1STNONRZ
PEDW_ADD_FLAG_14	Perinatal / Neonatal admission (Yes/No)	P00 – P96	ANY/1STNONRZ
PEDW_ADD_FLAG_15	Victimisation (Yes/No)	P96.1, T74, X85–Y34, Z04.0, Z04.5, Z04.8, Z60–Z63, Z65.3, Z72, Z74, Z76.1, Z76.2, Z81, Z86.5, Z91.6, Z91.8	ANY/1STNONRZ
PEDW_ADD_FLAG_16	Maltreatment (Yes/No)	T74, Y06, Y07	ANY/1STNONRZ
PEDW_ADD_FLAG_17	Assault (Yes/No)	X85 – Y03, Y04, Y05, Y08, Y09	ANY/1STNONRZ
PEDW_ADD_FLAG_18	Undetermined cause (Yes/No)	Y10 – Y34, Z04.0, Z04.5, Z04.8	ANY/1STNONRZ
PEDW_ADD_FLAG_19	Adverse social circumstances (Yes/No)	P96.1, Z60–Z63, Z65.3, Z72, Z74, Z76.1, Z76.2, Z81, Z86.5, Z91.6, Z91.8	ANY/1STNONRZ
PEDW_ADD_FLAG_20	Chemo/RT (Yes/No)	Z51.0-Z51.2, Z08.1, Z08.2, Z54.1, Z54.2	ANY/1STNONRZ
PEDW_CHP_01_FLAG	Chapter I: Certain infectious and parasitic diseases (Yes/No)	A00-B99	ANY/1STNONRZ
PEDW_CHP _02_FLAG	Chapter II: Neoplasms (Yes/No)	C00-D48	ANY/1STNONRZ
PEDW_CHP_03_FLAG	Chapter III: Diseases of the blood and blood-forming organs and certain disorders involving the immune	D50-D89	ANY/1STNONRZ

Variable required	Description	ICD-10 codes	Position
	mechanism (Yes/No)		
PEDW_CHP_04_FLAG	Chapter IV: Endocrine, nutritional and metabolic diseases (Yes/No)	E00-E90	ANY/1STNONRZ
PEDW_CHP_05_FLAG	Chapter V: Mental and behavioural disorders (Yes/No)	F00-F99	ANY/1STNONRZ
PEDW_CHP_06_FLAG	Chapter VI: Diseases of the nervous system (Yes/No)	G00-G99	ANY/1STNONRZ
PEDW_CHP_07_FLAG	Chapter VII: Diseases of the eye and adnexa (Yes/No)	H00-H59	ANY/1STNONRZ
PEDW_CHP_08_FLAG	Chapter VIII: Diseases of the ear and mastoid process (Yes/No)	H60-H95	ANY/1STNONRZ
PEDW_CHP_09_FLAG	Chapter IX: Diseases of the circulatory system (Yes/No)	I00-I99	ANY/1STNONRZ
PEDW_CHP_10_FLAG	Chapter X: Diseases of the respiratory system (Yes/No)	J00-J99	ANY/1STNONRZ
PEDW_CHP_11_FLAG	Chapter XI: Diseases of the digestive system (Yes/No)	K00-K93	ANY/1STNONRZ
PEDW_CHP_12_FLAG	Chapter XII: Diseases of the skin and subcutaneous tissue (Yes/No)	L00-L99	ANY/1STNONRZ
PEDW_CHP_13_FLAG	Chapter XIII: Diseases of the musculoskeletal system and connective tissue (Yes/No)	M00-M99	ANY/1STNONRZ

Variable required	Description	ICD-10 codes	Position
PEDW_CHP_14_FLAG	Chapter XIV: Diseases of the genitourinary system (Yes/No)	N00-N99	ANY/1STNONRZ
PEDW_CHP_15_FLAG	Chapter XV: Pregnancy, childbirth and the puerperium (Yes/No)	O00-O99	ANY/1STNONRZ
PEDW_CHP_16_FLAG	Chapter XVI: Certain conditions originating in the perinatal period (Yes/No)	P00-P96	ANY/1STNONRZ
PEDW_CHP_17_FLAG	Chapter XVII: Congenital malformations, deformations and chromosomal abnormalities (Yes/No)	Q00-Q99	ANY/1STNONRZ
PEDW_CHP_18_FLAG	Chapter XVIII: Symptoms, signs and abnormal clinical and laboratory findings, not elsewhere classified (Yes/No)	R00-R99	ANY/1STNONRZ
PEDW_CHP_19_FLAG	Chapter XIX: Injury, poisoning and certain other consequences of external causes (Yes/No)	S00-T98	ANY/1STNONRZ
PEDW_CHP_20_FLAG	Chapter XX: External causes of morbidity and mortality (Yes/No)	V01-Y98	ANY/1STNONRZ
PEDW_CHP_21_FLAG	Chapter XXI: Factors influencing health status and contact with health services (Yes/No)	Z00-Z99	ANY/1STNONRZ
PEDW_CHP_22_FLAG	Chapter XXII: Codes for special	U00-U85	ANY/1STNONRZ

Variable required	Description	ICD-10 codes	Position
	purposes (Yes/No)		
PEDW_OPR_FLAG_01	Paracentesis abdominis for ascites (Yes/No)	T46	ANY
PEDW_OPR_FLAG_02	Computed tomography of head (Yes/No)	U05	ANY
PEDW_OPR_FLAG_03	Fibreoptic endoscopic examination of upper gastrointestinal tract (Yes/No)	G43, G45	ANY
PEDW_OPR_FLAG_04	Magnetic resonance imaging (Yes/No)	U21	ANY
PEDW_OPR_FLAG_05	Fracture procedure (Yes/No)	V15, W19, W20, W24	ANY
PEDW_OPR_FLAG_06	Percutaneous biopsy of lesion of liver (Yes/No)	J13	ANY
PEDW_OPR_FLAG_07	Diagnostic endoscopic examination of lower bowel using fibreoptic sigmoidoscope (Yes/No)	H22, H25	ANY
PEDW_OPR_FLAG_08	Imaging of abdomen (Yes/No)	U08	ANY
PEDW_OPR_FLAG_09	Venesection (Yes/No)	X36	ANY
PEDW_OPR_FLAG_10	Ventilation support (Yes/No)	E85	ANY
PEDW_OPR_FLAG_11	Suture of skin of other site (Yes/No)	S42	ANY
PEDW_OPR_FLAG_12	Exploration of other skin of other site (Yes/No)	S57	ANY
PEDW_OPR_FLAG_13	Primary open reduction of fracture of	W26	ANY

**Variable required**	**Description**	**ICD-10 codes**	**Position**
	bone and extramed (Yes/No)		
PEDW_OPR_FLAG_14	Reduction of fracture of mandible (Yes/No)	V15	ANY
PEDW_OPR_FLAG_15	Blood transfusion (Yes/No)	X33	ANY
PEDW_OPR_FLAG_16	Iv infusion (Yes/No)	X29	ANY
PEDW_OPR_FLAG_17	Pleurocentesis (Yes/No)	T12	ANY
PEDW_OPR_FLAG_18	Chemo/RT (Yes/No)	X65,X67,X68, X70-X73	ANY

ANY: Any of the 14 coding position.

123RZ: The first three coding position or the fourth position if the first three coding positions contain only R or Z code (except of R78.0, Z50.2, Z71.4, Z72.1).

1STNONRZ: The first coding position, which contains neither R nor Z codes.
